# Editorial: Pharmacogenetics and Pharmacogenomics in Latin America: Ethnic Variability, New Insights in Advances and Perspectives: A RELIVAF-CYTED Initiative

**DOI:** 10.3389/fphar.2021.833000

**Published:** 2022-01-11

**Authors:** Patricia Esperón, Matías F. Martínez, María A. Redal, Alberto Lazarowski, Andrés López-Cortés, Nelson M. Varela, Luis A. Quiñones

**Affiliations:** ^1^ Molecular Genetics Laboratory, Clinical Biochemistry Department, School of Chemistry, Universidad de la República, Montevideo, Uruguay; ^2^ Latin American Network for the Implementation and Validation of Clinical Pharmacogenomics Guidelines (RELIVAF-CYTED), Madrid, Spain; ^3^ Laboratory of Chemical Carcinogenesis and Pharmacogenetics, Department of Basic-Clinical Oncology (DOBC), Faculty of Medicine, University of Chile, Santiago, Chile; ^4^ Genetic Division, Department of Medicine, INFIBIOC, Hospital de Clínicas José de San Martín, Buenos Aires University, Buenos Aires, Argentina; ^5^ Clinical Biochemistry Department, Hospital de Clínicas José de San Martín School of Pharmacy and Biochemistry, Institute for Research in Physiopathology and Clinical Biochemistry (INFIBIOC), University of Buenos Aires, Buenos Aires, Argentina

**Keywords:** pharmacogenetics, pharmacogenomics, CYTED, Latin America, precision medicine

**Editorial on the Research Topic**

**Pharmacogenetics and Pharmacogenomics in Latin America: Ethnic Variability, New Insights in Advances and Perspectives: A RELIVAF-CYTED Initiative**

## Introduction

Since the pharmacogenetics and pharmacogenomics (PGx) field started to rise, the information about the relationship between actionable genes, genotypes, and response to drugs has increased exponentially (Nicholson et al., 2021). There is evidence of the utility and impact of genetics in the choice of therapeutic regimens improving their effectiveness and safety (Arbitrio et al., 2021). Even some international efforts have created clinical guidelines that allow to implementation of pharmacogenomics in daily clinical practice. In addition to clinical outcomes, Economic benefits have been associated with the translation from “the bench to the bedside”.

Moreover, several major PGx expert organizations such as the Clinical Pharmacogenetics Implementation Consortium (CPIC, 2021) and the Dutch Pharmacogenetics Working Group (DPWG), provide gene-drug guidelines for actionable variants. In addition, Ubiquitous Pharmacogenomics (U-PGx, 2021), the Latin American Network for the Implementation and Validation of Pharmacogenomics Guidelines (RELIVAF-CYTED, 2021), and the Southeast Asian Pharmacogenomics Research Network (SEAPharm, Chumnumwat et al., 2019) have investigated pharmacotherapeutic recommendations guided by pharmacogenetics. In this respect, based on scientific evidence the Food and Drug Administration (FDA) has published a list of PGx biomarkers for drug labelling (FDA, 2021).

Even though high-quality research addresses the utility of implementing pharmacogenetics programs in clinical practice, most of this evidence comes from the United States or Europe. Moreover, commonly, it does not include the Latin American population, or when the guidelines do, it is considering as one big group. Some recently regional formed scientific societies (RELAGH, 2014; SOLFAGEM, 2021) and international efforts (RELIVAF-CYTED) are looking to shorten the region’s gap of evidence and information. In this respect, Latin America is a vast region with some characteristics that do not allow easy implementation of research made in other settings (Quiñones et al., 2014). It is one of the most genetically diverse areas having frequencies or polymorphisms not found in other regions. There is a lack of high-quality Latin American population-focused research about the relationship between specific genes and drug response, and, also, there is a lack of knowledge of frequencies. Altogether, there are many disadvantages to implementing pharmacogenetics in clinical practice in Latin America.

Sixteenth articles are included in this issue, eleven original/experimental research, two brief research reports, one review article, one case report, and one opinion, covering different and complementary aspects of the pharmacogenomic research in this region.

Workflows of data-driven modeling and model-driven experimentation have led to the development of *in silico* algorithms including pharmacogenomics data of disease risk at the patient-population level (Wolkenhauer et al., 2014). In this Research Topic four predictive models based on pharmacogenomics have been developed to identify patients who were suitable for preventive genotyping. Although the models must be validated with a larger number of patients and do not necessarily apply to all populations, they are a very good first approximation to predict the incidence of adverse effects among patients undergoing different therapies in Latin America. Miranda et al. proposed a model that included genetic polymorphisms in addition to clinical records, to predict the tamoxifen response in a population of 162 ER + breast cancer patients (Serral et al.) through an *in silico* approach, explored the druggable genes of two bacterial pathogens with a relevant impact in Latin America. The model proposed by Varela et al. was based on an integrated *in silico* analysis of breast and prostate cancer data genes. Finally, the algorithm created by Martinez et al. intended to predict the incidence of infections among patients under chemotherapy treatment.

The PGx of the immunosuppressive drug Tacrolimus (TAC) has been extensively studied, and according to the CPIC guidelines (Birdwell et al., 2015) an increase of starting dose for CYP3A5 expressers is recommended, followed by a therapeutic drug monitoring to guide dose adjustments. Thus, two manuscripts address the issue in Chilean kidney transplantation patients, one in children (Krall et al.), the other in an adult population (Contreras-Castillo et al.), for immunosuppressive treatment (cyclosporine and tacrolimus) after transplantation.

The antiretroviral treatment (ART) is generally not well tolerated and most patients present important adverse effects (ADR) that potentially limit treatment adherence or lead to this interruption (Saag et al., 2020). Poblete et al. retrospectively evaluated the UGT1A1*28 and CYP2B6 c.516G > T frequency and their influence on major ADRs in 67 adult HIV patients from Chile, as a starting point to validate in the nearest future CPIC guidelines in Latin America.

Two investigations referred to children with acute lymphoblastic leukemia from different angles. From Mexico, Gándara-Mireles et al. analyzed the frequency distribution and the association between the illness and the most common polymorphisms in ABCC1, NCF4, and CBR3 genes. The influence of TPMT-VNTR polymorphism on 6-MP related hematological toxicity was confirmed by Burgueño-Rodríguez et al. in 130 Uruguayan pediatric patients.

The studies performed in Duchenne Muscular Dystrophy (Luce et al.), cardiovascular disease (Gálvez et al.), and severe encephalopathy patients (Kravetz et al.) emphasized the importance of identifying both already known and novel variants to differential diagnosis and patient management. Luce et al. described the mutational spectrum of DMD gene in 400 Argentinian patients with a clinical diagnosis of dystrophinopathy. Gálvez et al. reported a significant association between APOB, APOE, and MTHFR polymorphisms and lipid levels, especially in women, among 193 healthy subjects from Chile. Identifying a genetic variant in KCNT1 channel in an Argentinian pediatric patient with a severe encephalopathy was crucial to include quinidine in the treatment regimen as an antiepileptic drug (Kravetz et al.).

Since discovering the non-coding RNA, its clinical relevance has become increasingly important. In particular, inter-individual variability in drug response, both in efficacy and toxicity, could be due to both, variation in miRNA gene sequences and circulating miRNA levels (Latini et al., 2019; Ubilla et al.) found an increase in miRNA-33b-5p levels in hypercholesterolemic patients under atorvastatin therapy and proposed this microRNA as a biomarker to follow the response to statins. Ruiz et al., worried about BCR-ABL1 tyrosine kinase inhibitor resistance in chronic myeloid leukemia patients, observed a global decrease of microRNA levels in resistant cells, founding a promiser field for future studies.

On other hand, the biobanks allow access to many well-classified, high-quality samples and establish the indispensable conditions for achieving reproducible research results (Coppola et al., 2019). Vargas and Cobar, express their opinion about creating biobanks and believe that the same requirements will be necessary to obtain pharmacogenetics information and efficient therapeutic responses in Latin America.

Barriers to PGx implementation include a lack of knowledge, training, and confidence among physicians to apply pharmacogenomic tests (Rigter et al., 2020). As such, Undurraga et al. reported on an anonymous online survey addressed to psychiatrists from Chile, observing a low acceptance of PGx tests, but a clear interest from psychiatrists in their potential incorporation into their clinical practice.

We proudly present this research topic which aims to address high-quality pharmacogenetic and pharmacogenomic research with a particular focus on the Latin American population and its needs. The main goal is to increase the information on the clinical implementation and the impact of pharmacogenetics in Latin American patients. Also, collecting experience and project the field in the region, looking for strategies and new perspectives. Furthermore, to potentiate the research among countries in the region.
